# Hepatitis B burden and population immunity in a high endemicity city – a geographically random household epidemiology study for evaluating achievability of elimination

**DOI:** 10.1017/S095026882300002X

**Published:** 2023-01-11

**Authors:** Ngai Sze Wong, Denise Pui Chung Chan, Chin Man Poon, Chin Pok Chan, Leonia Hiu Wan Lau, Eng-Kiong Yeoh, Shui Shan Lee

**Affiliations:** 1 Stanley Ho Centre for Emerging Infectious Diseases, The Chinese University of Hong Kong, Hong Kong, China; 2 JC School of Public Health and Primary Care, The Chinese University of Hong Kong, Shatin, Hong Kong, China; 3 Centre for Health Systems and Policy Research, The Chinese University of Hong Kong, Shatin, Hong Kong, China

**Keywords:** HBV elimination target, hepatitis B, population-based, prevalence, vaccination

## Abstract

This study aimed to provide reference for evaluating the achievability of hepatitis B virus (HBV) elimination in a high endemicity city with universal neonatal vaccination in place for over 30 years. Between September 2018 and October 2020, 2085 citizens from 1143 geographically random households in Hong Kong completed a questionnaire and had blood-testing for HBV markers (anti-HBs, HBsAg, anti-HBc, HBeAg). We evaluated the epidemiology and examined factors associated with HBV exposure, vaccination and chronic diseases. The proportion of households with HBsAg positive index participants was 9.2% (95% CI 7.5%–10.9%). The age- and sex-adjusted HBsAg prevalence was 6.3% (95% CI 5.3%–7.4%), compared to >10% in those born in 1960-1970 and among non-local born citizens, and <1% in people born after introduction of neonatal vaccination. Among 155 HBsAg positive participants, 59% were aware of their infection status with 10% on treatment and 10/150 (6.7%) HBeAg positive. More than 40% (872/2064) tested negative for both HBsAg and anti-HBs, contributed by the lack of immunity in older adults and the waning immunity of vaccines. Hong Kong has remained at high-intermediate HBV endemicity state. The moderate level of anti-HBs positivity and very low treatment coverage (10%) among HBsAg positive participants pose challenges for achieving the HBV elimination target.

## Introduction

Hepatitis B virus (HBV) infection carries the risk of chronicity, with threats of progression to cirrhosis, hepatocellular carcinoma (HCC) and associated mortality [1]. As of 2019, some 300 million people worldwide were living with chronic HBV infections [2]. In 2016, the World Health Organization (WHO) called for the elimination of viral hepatitis [3], the achievability of which hinges on the prevention of new infections and the reduction of chronic diseases. Prevention of HBV infections can be achieved largely through the implementation of universal vaccination, while prompt diagnosis and antiviral treatment of chronic HBV infection is needed in parallel to achieve elimination in the population [4]. Globally, the population burden of HBV infections varies geographically, as reflected by the seroprevalence of hepatitis B surface antigen (HBsAg) [5]. The HBsAg prevalence is highest at 5–10% among adult populations in sub-Saharan Africa and East Asia [3, 6], and lower in most industrialised countries. The variability highlighted the differences in the scale of the public health challenges posed to each city or country.

Since the early 1980s, many cities/countries in highly endemic Asia had developed vaccination programmes as a key strategy for reducing HBV population burden. After prioritising higher risk populations in the initial phase, universal vaccination of all newborns rapidly became the standard in many places in the Asia Pacific region [7–10]. With the universal neonatal vaccination programme in place for over 3 decades, it is anticipated that the prevalence of HBV infection, especially that of the younger adult population, should have fallen to a very low level. As for patients with chronic HBV infections, antiviral treatment has, in the past decade, become increasingly adopted in the region [11, 12]. Located in the Asia Pacific and physically linked with Mainland China, Hong Kong was one of the high endemicity jurisdictions with a high HBsAg prevalence of about 10% among healthy donors in the 1980s [6]. Similar to the timeline of neighbouring countries, hepatitis B vaccination was introduced in Hong Kong in 1982 [13], with universal vaccination provided to all newborns since 1988, a strategy subsequently strengthened by mop-up vaccination of pre- and primary school children [14]. (Supplementary Fig. 1) With a 3-dose schedule, vaccinations are given at birth, at 1 month and at 6 months of age. The HBV 3-dose vaccination coverage in children aged 2–5 were stable at above 99% each year from 2001 to 2018. [15]. Since 1980s, universal antenatal screening for HBsAg has been in place with above 99% coverage [16]. In 2020, the government launched an action plan to eliminate viral hepatitis with reference to the WHO's recommendations (https://www.hepatitis.gov.hk/english/action_plan/content.html). This study was designed to determine the current population burden of HBV infection in this previously high endemicity city of Hong Kong, following long history of universal vaccinations.

## Materials and methods

### Study subjects and study design

This is a territory-wide study with the recruitment of subjects from geographically random households, using a cluster sampling frame based on building groups in Hong Kong, the protocol of which has been published [17]. With informed consent, each household representative (index participant) was contacted for questionnaire completion and blood-taking arrangement. To compensate for the travel expenses a HKD25 voucher (USD1~HKD7.8) was given to each participant who had completed the study. Consent forms of participants aged below 18 were signed by their legal guardians. Ethical approval from the Joint Chinese University of Hong Kong – New Territories East Cluster Clinical Research Ethics Committee (CREC 2017.697) had been obtained.

The main outcome variables were HBsAg and anti-HBs test results (positive and negative). Independent variables included (i) socio-demographics – age, year of birth, gender, marital status, education level, ethnicity, place of birth, economic activity status; (ii) history of viral hepatitis testing, diagnosis and treatment, self-reported as HBV carriers and family history of infection; (iii) history of potential exposure to HBV including behavioural risk; and (iv) history of vaccination.

### Laboratory testing

All blood samples were collected through venesection, except for six children and two adults who had dried blood spots (DBS) collected because of difficulty with venesection. Due to low sensitivity and specificity, DBS test results were excluded from analysis in this study. Screening of serological markers of HBV (HBsAg, anti-HBs Ab and anti-HBc Ab) in each subject and testing of HBeAg in subjects tested HBsAg positive were performed by ELISA on an automated immunoassay system (MINI Vidas®, BioMérieux). All tests were performed and interpreted in accordance with the manufacturers' recommendations.

### Statistical analysis

We estimated the proportion of households testing HBsAg positive, with 95% confidence interval (CI) using binomial exact method. Birth year-gender specific prevalence of HBsAg in the population was estimated by selecting the smallest member ID of the household in the respective stratum. Sensitivity analysis was performed by selecting the youngest member in each stratum. Crude HBV prevalence was the proportion of participants with valid HBsAg test result testing positive. The prevalence was further adjusted by the distribution of population by gender and age groups from the government's Census and Statistics Department.

In the analyses, we examined factors associated with HBV exposure, vaccination coverage and chronic diseases. In evaluating the correlates of HBsAg positivity, bivariable and multivariable logistic regression models with age as a confounder were performed. To account for the association of HBsAg status of household members, a sub-analysis with age and HBsAg positivity of household members as confounders was performed after exclusion of single participant households. Among HBsAg positive participants, those who self-reported as HBV carriers at the time of survey were compared with others unaware in bivariable logistic regression models. For HBsAg negative participants, comparison was made between anti-HBs negative (undetectable immunity) and positive (detectable immunity) subjects, while among participants testing negative for both HBsAg and anti-HBs, factors associated with anti-HBc positive (inferring exposure) results were examined by performing bivariable and multivariable logistic regression models with age as a confounder. To examine the overall serologies, comparison was made between susceptible (negative for both HBsAg and anti-HBs), immune (anti-HBs positive, HBsAg negative), and chronically infected (HBsAg positive, anti-HBs negative) persons, after exclusion of cases with unclear status (HBsAg negative, anti-HBc positive, and anti-HBs negative) in multivariable multinominal logistic regression with variable of year of birth after 1990 as a confounder in SPSS25. Complete case analyses were performed.

## Results

### General characteristics of study population

Between September 2018 and October 2020, a total of 38 020 invitation letters were dispatched by post spanning over 2 years. We received 1497 (4%) household replies indicating consent to join the study. Ultimately, 1143 (76%) responding households completed the study, resulting in the enrolment of 2085 participants with a participants-to-household ratio of 1.82. About half (52%) of the recruited households had at least two members joining the study. Of all participants, 44% were male, with a median age of 54 (IQR = 39–63, range = 4–89 years old), almost all (99%) were ethnic Chinese and local permanent residents (98%) while 71% were born in Hong Kong (Supplementary Table S1). Comparing with population by-census data of 2016, a higher proportion of recruited participants were Chinese, female, aged 35 or above and with post-secondary education attainment (Supplementary Table S2). However, the distribution of recruited households by residential district was within 2% difference to that of residential building groups (Supplementary Table S3).

**Fig. 1.**
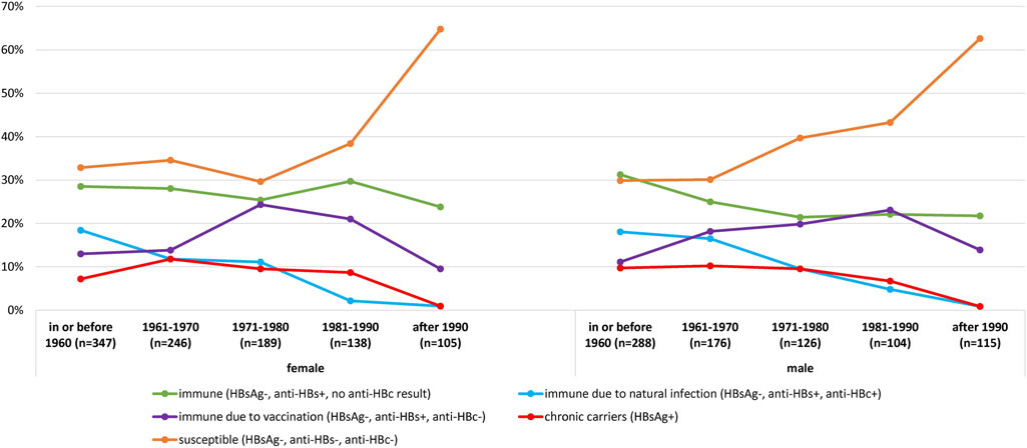
Distribution of participants' HBV serology by birth cohort and gender, after the exclusion of unclear status (HBsAg-, anti-HBs-, anti-HBc+) (selection for more than 1 member per household in age-gender strata: smallest member number).

By hepatitis B serology, the different proportions of participants who were chronic carriers (HBsAg + ), immune due to vaccination (HBsAg-, anti-HBs + ) and immune from natural infection (HBsAg-, anti-HBs + , anti-HBc + ) by birth cohort are shown in Figure 1. The youngest people (born after 1990) had the lowest proportion with HBsAg + and highest proportion with presumptive susceptibility to HBV infection because of absent anti-HBs. The proportion with natural immunity increased with age, while that from vaccination peaked for the birth cohort of 1981–1990 for male and 1971–1980 for female.

### Chronic hepatitis B infection

Overall, 9.2% (95% CI 7.5%–10.9%) of the index household participants tested HBsAg positive, while 12.8% (95% CI 10.8%–14.7%) of the households had participant testing positive (Supplementary Table S3). Among households with at least two members joining the study (*n* = 598), 16% had one or more member who tested HBsAg positive. Geographically, the proportion of households with index participants testing HBsAg positive varied by district (a total of 18 districts) (Supplementary Fig. 2), but the difference was not statistically significant (*χ*^2^ test, *P* = 0.2). Spatial statistical comparison could not be made with only 18 districts as geographic unit.

**Fig. 2.**
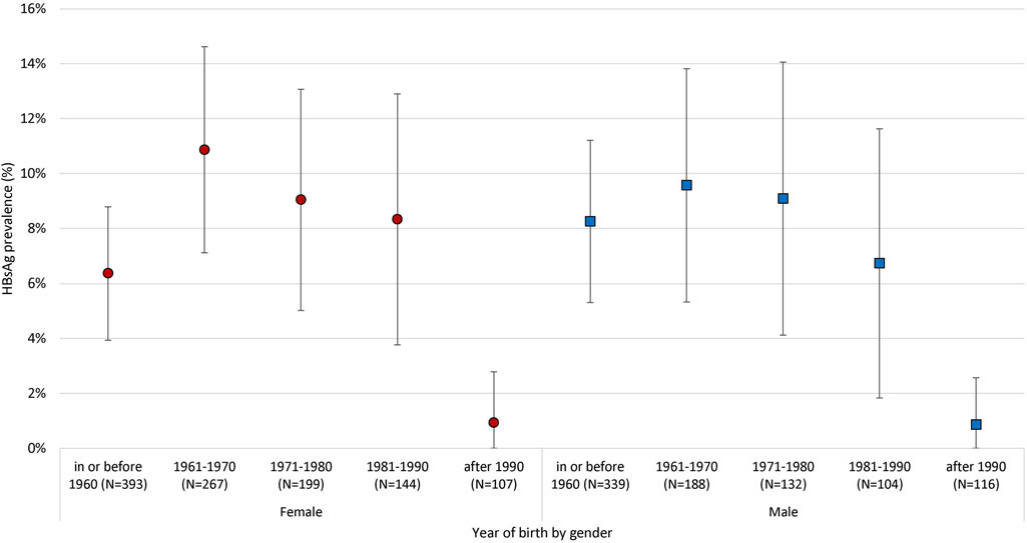
Birth year-gender stratified prevalence of HBsAg (selection for more than 1 member per household in age-gender strata: smallest member number).

Stratified by birth year group and gender, HBsAg prevalence was the highest among participants born in 1961–1970 for both female (10.9%, 95% CI 7.1%–14.6%) and male (9.6%, 95% CI 5.3%–13.8%) (Fig. 2). A declining prevalence was observed in both gender after this birth year stratum, with the lowest prevalence in participants born after 1990 (female at 0.9%, male at 0.8%). Sensitivity analysis was performed by selecting the youngest member in the birth year-gender strata, showing no difference in the count of HBsAg positive cases. On an individual level, 155/2076 participants tested HBsAg positive, giving a crude prevalence of 7.5% (95% CI 6.3%–8.6%), and age- and sex-adjusted prevalence of 6.3% (95% CI 5.3%–7.4%) (Supplementary Table S4). Comparing between subjects with HBsAg results, the proportion positive for HBsAg was higher among those born in Mainland China than Hong Kong (12% *vs.* 5%, odds ratio (OR) 0.40, 95% CI 0.29–0.57).

### The HBsAg positive population

There were altogether 155 (7%) HBsAg positive persons out of 2070 participants. Comparing with HBsAg negative participants, HBsAg positive participants were older (OR 1.01, 95% CI 1.004–1.03) or more likely born in or before 1990 (OR 11.38, 95% CI 2.80–46.21) (Table 1). Participants having attended local childhood immunisation programme (adjusted OR (aOR) 0.14, 95% CI 0.05–0.36), who had a post-secondary education level (aOR 0.59, 95% CI 0.41–0.86), born in Hong Kong (aOR 0.41, 95% CI 0.29–0.58), and being local residents (aOR 0.38, 95% CI 0.15–0.93) were less likely to be HBsAg positive after adjusting by age. All HBsAg positive subjects were ethnic Chinese, and none giving a history of illicit drug use or dialysis. In the sub-analysis, the same variables were significantly associated with HBsAg positive results after adjusting by age and by household member testing HBsAg positive, except for being local residents, history of fatty liver and number of lifetime sex partners which were insignificant factors (Supplementary Table S5).

**Table 1.** Comparison between HBsAg negative (*N* = 1915) and positive participants (*N* = 155)

	HBsAg−ve		HBsAg + ve		Odds ratio (OR)		Adjusted OR^a^	
	*n*	%	*n*	%	OR	(95% CI)	aOR	(95% CI)
Socio-demographics								
Gender, *N* = 2070								
Female	1077	92%	88	8%	ref		ref	
Male	838	93%	67	7%	0.98	(0.7–1.36)	0.98	(0.7–1.36)
Median age (IQR), *N* = 2070	54	(38–63)	56	(45–63)	1.01*	(1.004–1.03)	/	
Year of birth								
In or before 1990	1667	92%	153	8%	11.38*	(2.80–46.21)	/	
After 1990	248	99%	2	1%	ref			
Ethnicity, *N* = 2058								
Non-Chinese	13	100%	0	0%				
Chinese	1891	92%	154	8%	/		/	
Hong Kong permanent residents, *N* = 2057								
No	35	85%	6	15%	ref		ref	
Yes	1869	93%	147	7%	0.46	(0.19–1.11)	0.38*	(0.15–0.93)
Born in Hong Kong, *N* = 2069								
No	521	87%	76	13%	ref		ref	
Yes	1393	95%	79	5%	0.39*	(0.28–0.54)	0.41*	(0.29–0.58)
Ever married, *N* = 1993								
No	473	95%	24	5%	ref		ref	
Yes	1366	91%	130	9%	1.88*	(1.2–2.94)	1.76*	(1.06–2.92)
Education level, *N* = 2063								
Secondary and below	1076	91%	109	9%	ref		ref	
Post-secondary	838	95%	46	5%	0.54*	(0.38–0.77)	0.59*	(0.41–0.86)
History of hepatitis or other liver diseases								
Liver diseases, *N* = 2065								
No	1799	93%	134	7%	ref		ref	
Yes	112	85%	20	15%	2.40*	(1.44–3.98)	2.22*	(1.33–3.69)
Fatty liver, *N* = 2070								
No	1812	93%	138	7%	ref		ref	
Yes	103	86%	17	14%	2.17*	(1.26–3.72)	2.00*	(1.16–3.45)
Cirrhosis, *N* = 2070								
No	1913	93%	154	7%	ref		ref	
Yes	2	67%	1	33%	6.21	(0.56–68.88)	5.7	(0.51–63.3)
Liver cancer, *N* = 2070								
No	1913	93%	153	7%	ref		ref	
Yes	2	50%	2	50%	12.50*	(1.75–89.38)	11.36*	(1.56–82.45)
Family member with HBV infection, *N* = 2036								
No	1537	95%	89	5%	ref		ref	
Yes	341	85%	61	15%	3.09*	(2.19–4.37)	3.38*	(2.38–4.81)
Not sure	6	75%	2	25%	5.76*	(1.15–28.93)	5.14*	(1.02–25.95)
Household member testing HBsAg positive in this study, *N* = 1527								
No	1280	93%	89	7%	ref		ref	
Yes	142	90%	16	10%	1.62	(0.93–2.84)	1.78*	(1.01–3.13)
Community exposure risk								
History of illicit drug use, *N* = 1997								
No	1841	92%	154	8%				
Yes	2	100%	0	0%	/		/	
Sex experience, *N* = 2004								
No	285	96%	13	4%	ref		ref	
Yes	1564	92%	142	8%	1.99*	(1.11–3.56)	1.8	(0.98–3.29)
No. of lifetime sex partners, *N* = 1952								
None	285	96%	13	4%	ref		ref	
1	1060	92%	94	8%	1.94*	(1.07–3.52)	1.69	(0.9–3.17)
2 to 5	386	91%	37	9%	2.10*	(1.1–4.03)	1.98*	(1.03–3.82)
6 to 10	49	88%	7	13%	3.13*	(1.19–8.24)	2.96*	(1.12–7.81)
11 or more	21	100%	0	0%	/		/	
Risk exposure in the healthcare setting								
Frequency of receiving intravenous injection, *N* = 2039								
Never	1218	94%	78	6%	ref		ref	
Ever	668	90%	75	10%	1.75*	(1.26–2.44)	1.70*	(1.22–2.37)
Frequency of blood transfusion, *N* = 2028								
Never	1634	93%	132	7%	ref		ref	
Ever	240	92%	22	8%	1.13	(0.71–1.82)	1.03	(0.64–1.66)
History of dialysis, *N* = 2030								
Never	1872	92%	154	8%				
Ever	4	100%	0	0%	/		/	
History of surgery, *N* = 2038								
Never	1023	93%	81	7%	ref		ref	
Ever	862	92%	72	8%	1.05	(0.76–1.47)	0.94	(0.67–1.32)
Vaccination history								
Self-reported HBV vaccination, *N* = 2058								
No	1183	89%	143	11%	ref		ref	
Yes	720	98%	12	2%	0.14*	(0.08–0.25)	0.14*	(0.08–0.26)
HBV childhood immunisation in Hong Kong^b^, *N* = 2070								
Unlikely	1563	91%	149	9%	ref		ref	
Likely	352	98%	6	2%	0.18*	(0.08–0.41)	0.14*	(0.05–0.36)

a age as the confounder in multivariable logistic regression.

b Born in Hong Kong in or after 1984 or migrate to Hong Kong at the age 12 or below.

*P < 0.05.

Only 59% (91/155) of the HBsAg positive participants were aware of their status and self-reported as HBV carriers (referred as ‘known carriers’ in this study) (Table 2). Among known carriers, 11% (9/80) were diagnosed within 5 years prior to the survey, while 34% (27/79) have ever been in care, and 10% (12/88) on antiviral treatment. Ten out of 150 HBsAg positive participants (6.7%, 95% CI 2.6%–10.7%) were HBeAg positive: four male, with median age of 47.5 (IQR = 36–54 years old), six being known carriers, and none in HBV care or treatment. There was no difference in HBeAg positive status and age between known carriers and those unaware of their infection status (unknown carriers). Known carriers were more likely to have family member with HBV infection (OR 2.34, 95% CI 1.17–4.65), and past history of surgery (OR 3.17, 95% CI 1.41–7.10; Never as reference). A lower proportion of ‘known carriers’ had household members who tested HBsAg positive (OR 0.28, 95% CI 0.09–0.88).

**Table 2.** Characteristics of participants who tested HBsAg positive in the study (*n* = 155) with comparison between known carriers (*n* = 91) and unknown carriers who were unaware of their status (*n* = 64)

	Overall		Unknown carrier		Known carrier		Odds ratio (OR)	
	*n* (%)		*n* %		*n* %		OR lower	upper
Socio-demographics								
Gender								
Female	88 (57%)	36	56%	52	57%	ref		
Male	67 (43%)	28	44%	39	43%	0.96	0.51	1.84
Median age (IQR)	56 (45–63)	57	42.75–62.75	55	46–63	1.00	0.98	1.03
Ethnicity, *n* = 154								
Non-Chinese	0 (0%)	0	0%	0	0%			
Chinese	154 (100%)	63	100%	91	100%	/		
Hong Kong permanent residents, *n* = 153								
No	6 (4%)	5	8%	1	1%	ref		
Yes	147 (96%)	57	92%	90	99%	7.89	0.90	69.32
Born in Hong Kong								
No	76 (49%)	34	53%	42	46%	ref		
Yes	79 (51%)	30	47%	49	54%	1.32	0.70	2.51
Ever married, *n* = 154								
No	24 (16%)	9	14%	15	16%	ref		
Yes	130 (84%)	54	86%	76	84%	0.84	0.34	2.07
Education level								
Secondary and below	109 (70%)	49	77%	60	66%	ref		
Post-secondary	46 (30%)	15	23%	31	34%	1.69	0.82	3.48
History of hepatitis or other liver diseases								
Liver diseases, *n* = 154								
No	134 (87%)	58	92%	76	84%	ref		
Yes	20 (13%)	5	8%	15	16%	2.29	0.79	6.66
Fatty liver								
No	138 (89%)	60	94%	78	86%	ref		
Yes	17 (11%)	4	6%	13	14%	2.50	0.78	8.06
Cirrhosis								
No	154 (99%)	63	98%	91	100%			
Yes	1 (1%)	1	2%	0	0%	/		
Liver cancer								
No	153 (99%)	64	100%	89	98%			
Yes	2 (1%)	0	0%	2	2%	/		
Family member with HBV infection, *n* = 152								
No	89 (59%)	44	71%	45	50%	ref		
Yes	61 (40%)	18	29%	43	48%	2.34*	1.17	4.65
Not sure	2 (1%)	0	0%	2	2%	/		
Household member testing HBsAg positive in this study								
No	89 (85%)	34	76%	55	92%	ref		
Yes	16 (15%)	11	24%	5	8%	0.28*	0.09	0.88
Community exposure risk								
History of illicit drug use, *n* = 154								
No	154 (100%)	63	100%	91	100%			
Yes	0 (0%)	0	0%	0	0%	/		
Sex experience								
No	13 (8%)	4	6%	9	10%	ref		
Yes	142 (92%)	60	94%	82	90%	0.61	0.18	2.07
No. of lifetime sex partners, *n* = 151								
None	13 (9%)	4	6%	9	10%	ref		
1	94 (62%)	38	61%	56	63%	0.65	0.19	2.28
2 to 5	37 (25%)	19	31%	18	20%	0.42	0.11	1.61
6 to 10	7 (5%)	1	2%	6	7%	2.67	0.24	30.07
11 or more	0 (0%)	0	0%	0	0%			
Risk exposure in the healthcare setting								
Frequency of receiving intravenous injection, *n* = 153								
Never	78 (51%)	36	58%	42	46%	ref		
Once	31 (20%)	10	16%	21	23%	1.80	0.75	4.32
More than once	44 (29%)	16	26%	28	31%	1.50	0.70	3.20
Regular	0 (0%)	0	0%	0	0%	/		
Frequency of blood transfusion, *n* = 154								
Never	132 (86%)	55	87%	77	85%	ref		
Never	22 (14%)	8	13%	14	15%	1.25	0.49	3.18
History of dialysis, *n* = 154								
Never	154 (100%)	63	0%	91	0%			
Ever	0 (0%)	0	0%	0	0%	/		
History of surgery, *n* = 153								
Never	81 (53%)	41	66%	40	44%	ref		
Ever	72 (47%)	21	34%	51	56%	2.49*	1.27	4.86

*P < 0.05.

### Population immunity from HBV infection

Overall 1915 (93%) tested negative for HBsAg. The proportion of participants with self-reported history of vaccination by year of birth was: After 1990: 57%; 1981–1990: 56%; 1971–1980: 38%; 1961–1970: 33%; before 1960: 22%. Serologically, a total of 1037 (50%) participants tested negative for HBsAg (HBsAg-) and positive for anti-HBs (anti-HBs + ) (Supplementary Table S6), whose characteristics were compared with 872 anti-HBs negative (HBsAg- anti-HBs-) participants (Table 3). The median age of all HBsAg- individuals was 54 (IQR = 39–63 years old), with an older age for participants who tested anti-HBs + (OR 1.01, 95% CI 1.003–1.01). Participants born in 1990 or before (OR ranging between 2.04 for ≤1950 and 2.68 for 1971–1980; with >1990 as reference) were more likely to test for anti-HBs. Adjusted by age, locally born participants (aOR 0.66, 95% CI 0.53–0.81), local permanent residents (aOR 0.36, 95% CI 0.17–0.78), and those having attended local childhood immunisation programme (aOR 0.54, 95% CI 0.38–0.78) were less likely to test anti-HBs + . Participants with self-reported history of HBV vaccination (aOR 2.70, 95% CI 2.19–3.32) were more likely to be anti-HBs + . Overall comparisons between immune and susceptible, and between immune and chronically infected are shown in Supplementary Table S7.

**Table 3.** Characteristics of HBsAg negative participants (*n* = 1909) with comparison between anti-HBs negative (*n* = 872) and positive (*n* = 1037) cases

	Anti-HBs negative		Anti-HBs positive		Bivariable logistic regression		Multivariable logistic regression^a^	
	*n*	%	*n*	%	crude OR	95% CI	aOR	95% CI
Socio-demographics								
Gender, *N* = 1909								
Female	485	56%	587	57%	Ref			
Male	387	44%	450	43%	0.96	0.8–1.15	0.96	0.8–1.16
Median age (IQR), *N* = 1909	53	35–63	54	42–63	1.01*	1.003–1.01	/	
Year of birth group								
In or before 1960	305	35%	389	38%	2.36*	1.74–3.19	/	
1961–1970	177	20%	241	23%	2.52*	1.82–3.49		
1971–1980	125	14%	181	17%	2.68*	1.89–3.79		
1981–1990	106	12%	140	14%	2.44*	1.7–3.51		
After 1990	159	18%	86	8%	Ref			
Ethnicity, *N* = 1898								
Non-Chinese	9	1%	3	0%	Ref			
Chinese	857	99%	1029	100%	3.60	0.97–13.35	3.56	0.96–13.24
Hong Kong permanent residents, *N* = 1899								
No	9	1%	26	3%	Ref			
Yes	858	99%	1006	97%	0.41*	0.19–0.87	0.36*	0.17–0.78
Born in Hong Kong, *N* = 1908								
No	194	22%	325	31%	Ref			
Yes	677	78%	712	69%	0.63*	0.51–0.77	0.66*	0.53–0.81
Ever married, *N* = 1833								
No	260	31%	211	21%	Ref			
Yes	569	69%	793	79%	1.72*	1.39–2.12	1.73*	1.35–2.22
Education level, *N* = 1902								
Secondary and below	486	56%	582	56%	Ref			
Post-secondary	383	44%	451	44%	0.98	0.82–1.18	1.08	0.89–1.31
Any member tested HBsAg + ve, including oneself, *N* = 1909								
No	814	93%	953	92%	Ref			
Yes	58	7%	84	8%	1.24	0.87–1.75	1.33	0.94–1.89
Anti-HBc + ve, *N* = 1388								
No	720	83%	303	58%	Ref			
Yes	144	17%	221	42%	3.65*	2.84–4.68	3.76*	2.88–4.92
HBV vaccination status								
Self-report HBV vaccination, *N* = 1897								
No	625	72%	556	54%	Ref			
Yes	241	28%	475	46%	2.22*	1.83–2.69	2.70*	2.19–3.32
HBV childhood immunisation in Hong Kong^b^, *N* = 1909								
Unlikely	674	77%	886	85%	Ref			
Likely	198	23%	151	15%	0.58*	0.46–0.73	0.54*	0.38–0.78

a adjusted by age in multivariable logistic regression.

b Born in Hong Kong in or after 1984 or migrate to Hong Kong at the age 12 or below.

*P < 0.05.

### Natural infection *vs.* HBV vaccination

Excluding the 7.5% HBsAg positive participants with chronic HBV infection, some 17% (144/864) of those who tested negative for both HBsAg and anti-HBs, were anti-HBc positive, reflecting natural infection following exposure. Anti-HBc positive participants were older than anti-HBc negative counterparts (median age 64 *vs.* 49, OR 1.08, 95% CI 1.06–1.09) (Table 4). They were less likely to be born locally (aOR 0.27, 95% CI 0.18–0.41), and have a post-secondary education level (aOR 0.55, 95% CI 0.35–0.87), but more likely to be married (aOR 2.24, 95% CI 1.12–4.48) after adjusting by age, as compared to anti-HBc negative participants.

**Table 4.** Characteristics of participants who tested negative for both HBsAg and anti-HBs (*n* = 872), with comparison by anti-HBc test result (720 anti-HBc −ve, 144 anti-HBc + ve)

	HBsAg negative and anti-HBs negative		Anti-HBc negative		Anti-HBc positive		*N*	Bivariable logistic regression		Multivariable logistic regression^a^	
	*n*	%	*n*	%	*n*	%		crude OR	95% CI	aOR	95% CI
Socio-demographics											
Gender											
Female	485	56%	403	56%	77	53%	480	Ref			
Male	387	44%	317	44%	67	47%	384	1.11	0.77–1.58	1.10	0.74–1.61
Median age (IQR)	53	35–63	49	31–61	64	57–70	864	1.08*	1.06–1.09	/	
Year of birth group											
In or before 1960	305	35%	207	29%	94	65%	301	Ref		/	
1961–1970	177	20%	144	20%	31	22%	175	0.47*	0.30–0.75		
1971–1980	125	14%	109	15%	15	10%	124	0.30*	0.17–0.55		
1981–1990	106	12%	102	14%	4	3%	106	0.09*	0.03–0.24		
After 1990	159	18%	158	22%	0	0%	158	/			
Ethnicity											
Non-Chinese	9	1%	9	1%	0	0%	9				
Chinese	857	99%	706	99%	143	100%	849	/		/	
Hong Kong permanent residents											
No	9	1%	7	1%	2	1%	9	Ref			
Yes	858	99%	710	99%	140	99%	850	0.69	0.14–3.36	0.64	0.11–3.61
Born in Hong Kong											
No	194	22%	116	16%	75	52%	191	Ref			
Yes	677	78%	603	84%	69	48%	672	0.18*	0.12–0.26	0.27*	0.18–0.41
Ever married											
No	260	31%	246	36%	11	8%	257	Ref			
Yes	569	69%	431	64%	133	92%	564	6.90*	3.66–13.02	2.24*	1.12–4.48
Education level											
Secondary and below	486	56%	370	52%	112	78%	482	Ref			
Post-secondary	383	44%	347	48%	32	22%	379	0.30*	0.2–0.46	0.55*	0.35–0.87
Any member tested HBsAg + ve											
No	814	93%	666	93%	140	97%	806	Ref			
Yes	58	7%	54	8%	4	3%	58	0.35*	0.13–0.99	0.45	0.15–1.37
HBV vaccination status											
Self-report HBV vaccination											
No	625	72%	496	69%	123	87%	619	Ref			
Yes	241	28%	220	31%	19	13%	239	0.35*	0.21–0.58	0.61	0.35–1.04
HBV childhood immunisation in Hong Kong^b^											
Unlikely	674	77%	523	73%	144	100%	667				
Likely	198	23%	197	27%	0	0%	197	/		/	

a adjusted by age in multivariable logistic regression.

b Born in Hong Kong in or after 1984 or migrate to Hong Kong at the age 12 or below.

*P < 0.05.

## Discussion

In this population-level HBV study conducted in 2020, the crude HBsAg prevalence was 7.5%, which places Hong Kong in the high-intermediate endemicity category by epidemiologic state [5]. With age/sex adjustment, the population prevalence was 6.5%, which is slightly lower than that of 7.2% in an earlier study of 2015/2016 [18], further distancing from the 8.8% in a population survey conducted 20 years ago in 2001 [15], and the 9.5% prevalence among blood donors some 30–40 years ago [13]. Our study provided evidence that the HBV population burden has been falling over the last decades. The family clustering and the higher prevalence in older population was similar to the pattern observed before the universal vaccination programme was in place [13]. The universal neonatal vaccination programme and the interventions for reducing mother-to-child transmission (MTCT) of HBV have contributed to the continued reduction of new infections. The MTCT strategy includes hepatitis B immunoglobulin prophylaxis for newborns of HBsAg positive mothers, and more recently, antivirals to mothers with high HBV viral load and post-vaccination serology testing. The neonatal vaccination and MTCT programmes have contributed to a dramatic fall of HBsAg prevalence to below 1% for people age below 30. Nevertheless, the overall reduction of HBV carriage at population level had occurred only very gradually, the pattern of which was seen in the results of a population cohort in Taiwan where the HBsAg prevalence was similarly high in the past [19].

With a median age of 56, the HBsAg positive persons in the current study were not just older but that over 40% were unaware of their infection status, of which <20% had been on any form of HBV therapy. Taking HBeAg positivity as a marker of chronic active hepatitis [20], this subpopulation accounted for less than 10% of the HBsAg positive carriers, but none was on any treatment. The estimated half a million persons with positive HBsAg status in Hong Kong is a cause for concern. Epidemiologically, it appears unlikely that the burden will decrease precipitously given the pace of population ageing and the small proportion of HBsAg positive patients on any form of antiviral therapy [21].

In this study, population immunity as inferred from the presence of anti-HBs could be shown in only 50% of the local residents after excluding all HBsAg positive carriers. The low self-reported history of vaccination was related to the uncertainty of participants' knowledge of immunisation status which was not always available in health records. With a high vaccination coverage of over 90% in Hong Kong [15], protective immunity can be demonstrated serologically in only 35%. This suggested the lack of immunity in residents born before implementation of the universal vaccination programme and the waning of vaccination effects over time, [22, 23] which was consistent with a previous local study's findings [24]. As our recruitment has targeted adults in households, we may not have included a high proportion of children and adolescents with higher anti-HBs following vaccination. There were a higher proportion of anti-HBc negative persons among the younger anti-HBs negative adults, suggesting the absence of natural infection following vaccination. While anamnestic responses could be elicited 20 years or more following full vaccination in childhood [25, 26], the absence of anti-HBs in such cases does pose a challenge in assessing population immunity. On the other hand, natural HBV infection might have occurred in 17% of the anti-HBs negative persons, as shown by the presence of anti-HBc. The phenomenon of isolated anti-HBc is however a technical challenge. Apart from being a possibly false positive result, it may imply occult HBV infection or occurrence during the window period of acute HBV infection and the late-stage infection after anti-HBs has fallen to undetectable levels [27].

Our study carries a few limitations. First, the return rate was low (4%), and the recruitment rate was slowed by the COVID-19 epidemic. Second, self-selection bias might have arisen as we did not exclude known infected individuals and their household members, who might be attracted to join the study for checking their status. Conversely, some might have self-deferred as they have already known their infection status. Third, there might be recall bias in survey items relating to history of vaccination and potential risk exposure, while some participants were uncertain about their vaccination history in childhood. Also, we have collected minimal data for epidemiological purpose and had not covered clinical histories.

In conclusion, the introduction of universal HBV vaccination in Hong Kong has contributed to the reduction of population burden with evolvement from a high to high-intermediate endemicity status. Whereas half the population has demonstrable immuno-protection against HBV infection, the prevalence of chronic infection has fallen only slightly. The continuation of high coverage universal neonatal vaccination programme would undoubtedly further reduce the HBV burden in the coming decades. The low prevalence of HBsAg and anti-HBc among the vaccinated people, the continuing presence of anti-HBs alone or with anti-HBc supports the hypothesis of an anamnestic response and for the proposition of not giving booster doses in late adult life. Booster HBV vaccination remains controversial [28], which is generally not recommended by international guidelines including that of the WHO [29], though single booster dose administration has been advocated [30]. With the high number of HBsAg positive older adults, the WHO target of providing treatment to 80% eligible hepatitis B patients by 2030 [3] would be a challenge. HBV infection in elderly people is a neglected issue the importance of which is poorly appreciated in public health context [31]. Innovative approach to population screening in conjunction to practical strategy for linkage to care is yet to be developed.

## Data Availability

The data that support the findings of this study are available from the corresponding author, SS Lee, upon reasonable request.

## Supplementary material

For supplementary material accompanying this paper visit http://doi.org/10.1017/S095026882300002X. click here to view supplementary material
