# Hepatitis B burden and population immunity in a high endemicity city – a geographically random household epidemiology study for evaluating achievability of elimination – ERRATUM

**DOI:** 10.1017/S0950268823000341

**Published:** 2023-03-16

**Authors:** Ngai Sze Wong, Denise Pui Chung Chan, Chin Man Poon, Chin Pok Chan, Leonia Hiu Wan Lau, Eng-Kiong Yeoh, Shui Shan Lee

**Affiliations:** 1 Stanley Ho Centre for Emerging Infectious Diseases, The Chinese University of Hong Kong, Hong Kong, China; 2 JC School of Public Health and Primary Care, The Chinese University of Hong Kong, Shatin, Hong Kong, China; 3 Centre for Health Systems and Policy Research, The Chinese University of Hong Kong, Shatin, Hong Kong, China

There is a misalignment of Table 2 in the original article published. Please refer to the corrected version of Table 2 below.

**Table 2.** Characteristics of participants who tested HBsAg positive in the study (n=155) with comparison between known carriers (n=91) and unknown carriers who were unaware of their status (n = 64)

	Overall	unknown carrier		known carrier		Odds Ratio (OR)		
	n (%)	n	%	n	%	OR	lower	upper
**Socio-demographics**								
Gender								
Female	88 (57%)	36	56%	52	57%	ref		
Male	67 (43%)	28	44%	39	43%	0.96	0.51	1.84
Median age (IQR)	56 (45-63)	57	42.75-62.75	55	46-63	1.00	0.98	1.03
Ethnicity, n=154								
Non-Chinese	0 (0%)	0	0%	0	0%			
Chinese	154 (100%)	63	100%	91	100%	/		
Hong Kong permanent residents, n=153								
No	6 (4%)	5	8%	1	1%	ref		
Yes	147 (96%)	57	92%	90	99%	7.89	0.90	69.32
Born in Hong Kong								
No	76 (49%)	34	53%	42	46%	ref		
Yes	79 (51%)	30	47%	49	54%	1.32	0.70	2.51
Ever married, n=154								
No	24 (16%)	9	14%	15	16%	ref		
Yes	130 (84%)	54	86%	76	84%	0.84	0.34	2.07
Education level								
Secondary and below	109 (70%)	49	77%	60	66%	ref		
Post-secondary	46 (30%)	15	23%	31	34%	1.69	0.82	3.48
**History of hepatitis or other liver diseases**								
Liver diseases, n=154								
No	134 (87%)	58	92%	76	84%	ref		
Yes	20 (13%)	5	8%	15	16%	2.29	0.79	6.66
Fatty liver								
No	138 (89%)	60	94%	78	86%	ref		
Yes	17 (11%)	4	6%	13	14%	2.50	0.78	8.06
Cirrhosis								
No	154 (99%)	63	98%	91	100%			
Yes	1 (1%)	1	2%	0	0%	/		
Liver cancer								
No	153 (99%)	64	100%	89	98%			
Yes	2 (1%)	0	0%	2	2%	/		
Family member with HBV infection, n=152								
No	89 (59%)	44	71%	45	50%	ref		
Yes	61 (40%)	18	29%	43	48%	2.34*	1.17	4.65
Not sure	2 (1%)	0	0%	2	2%	/		
Household member testing HBsAg positive in this study								
No	89 (85%)	34	76%	55	92%	ref		
Yes	16 (15%)	11	24%	5	8%	0.28*	0.09	0.88
**Community exposure risk**								
History of illicit drug use, n=154								
No	154 (100%)	63	100%	91	100%			
Yes	0 (0%)	0	0%	0	0%	/		
Sex experience								
No	13 (8%)	4	6%	9	10%	ref		
Yes	142 (92%)	60	94%	82	90%	0.61	0.18	2.07
No. of lifetime sex partners, n=151								
None	13 (9%)	4	6%	9	10%	ref		
1	94 (62%)	38	61%	56	63%	0.65	0.19	2.28
2 to 5	37 (25%)	19	31%	18	20%	0.42	0.11	1.61
6 to 10	7 (5%)	1	2%	6	7%	2.67	0.24	30.07
11 or more	0 (0%)	0	0%	0	0%			
**Risk exposure in the healthcare setting**								
Frequency of receiving intravenous injection, n=153								
Never	78 (51%)	36	58%	42	46%	ref		
Once	31 (20%)	10	16%	21	23%	1.80	0.75	4.32
More than once	44 (29%)	16	26%	28	31%	1.50	0.70	3.20
Regular	0 (0%)	0	0%	0	0%	/		
Frequency of blood transfusion, n=154								
Never	132 (86%)	55	87%	77	85%	ref		
Ever	22 (14%)	8	13%	14	15%	1.25	0.49	3.18
History of dialysis, n=154								
Never	154 (100%)	63	0%	91	0%			
Ever	0 (0%)	0	0%	0	0%	/		
History of surgery, n=153								
Never	81 (53%)	41	66%	40	44%	ref		
Ever	72 (47%)	21	34%	51	56%	2.49*	1.27	4.86

# Born in Hong Kong in or after1984, or migrate to Hong Kong at the age 12 or below

*p<0.05

The publisher apologizes for the mistake.
