# Supplementary material for: Hepatitis B burden and population immunity in a high endemicity city – a geographically random household epidemiology study for evaluating achievability of elimination
Source: Epidemiol Infect. 2023 Jan 11;151:e22. doi: 10.1017/S095026882300002X (PMC9990397; doi:10.1017/S095026882300002X)

## Supplementary file

### Supplementary Table S1. General characteristics of participants (n=2085)

| | n | % |
|---|---|---|
| Gender | | |
| Female | 1175 | 56% |
| Male | 910 | 44% |
| Median age (IQR) | 54 | 39-63 |
| Year of birth | | |
| In or before 1950 | 224 | 11% |
| 1951-1960 | 527 | 25% |
| 1961-1970 | 467 | 22% |
| 1971-1980 | 339 | 16% |
| 1981-1990 | 267 | 13% |
| After 1990 | 261 | 13% |
| Ethnicity (n=2073) | | |
| Non-Chinese | 13 | 1% |
| Chinese | 2060 | 99% |
| Hong Kong permanent residents (n=2072) | | |
| No | 41 | 2% |
| Yes | 2031 | 98% |
| Born in Hong Kong (n=2084) | | |
| No | 598 | 29% |
| Yes | 1486 | 71% |
| Marital status | | |
| Never married | 500 | 24% |
| Widowed | 104 | 5% |
| Separated | 7 | 0.3% |
| Divorced | 148 | 7% |
| Married | 1240 | 60% |
| NA, 15 or below | 82 | 4% |
| Education level (n=2078) | | |
| Secondary and below | 1192 | 57% |
| Post-secondary | 886 | 43% |
| Economically active# (n=1997) | | |
| No | 844 | 42% |
| Yes | 1153 | 58% |

#economically active refers to employer, employee, self-employed, unpaid family worker

**Supplementary Table S2. Characteristics comparison among all participants, household representatives, and census population in 2016**

| | All participants, n=2085 (%) | Household representatives, n=1143 (%) | Population by-census 2016 (%)# |
|---|---|---|---|
| **Gender** | | | |
| Female | 56% | 55% | 52% |
| Male | 44% | 45% | 48% |
| **Age group (yo)** | | | |
| 0-14 | 3% | 0% | 12% |
| 15-34 | 16% | 9% | 25% |
| 35-64 | 60% | 67% | 47% |
| >64 | 21% | 24% | 17% |
| **Ethnicity** | | | |
| Non-Chinese | 1% | 0.4% | 4% |
| Chinese | 99% | 99.6% | 96% |
| **Education level&** | | | |
| Primary and below | 11% | 13% | 21% |
| Secondary | 45% | 45% | 46% |
| Post-secondary | 44% | 42% | 33% |

#population excluding foreign domestic helpers; data source: Census and Statistics Department, Hong Kong SAR Government.
&highest level of educational attainment for individuals aged 15 or above

**Supplementary Table S3. General characteristics of households (n=1143)**

| | n | % | % of building groups in the District |
|---|---|---|---|
| District, n=1142 | | | |
| A | 43 | 4% | 4% |
| B | 16 | 1% | 3% |
| C | 118 | 10% | 9% |
| D | 38 | 3% | 4% |
| E | 62 | 5% | 5% |
| F | 99 | 9% | 6% |
| G | 77 | 7% | 6% |
| H | 79 | 7% | 7% |
| J | 102 | 9% | 10% |
| K | 39 | 4% | 4% |
| L | 62 | 5% | 7% |
| M | 60 | 5% | 7% |
| N | 51 | 4% | 3% |
| P | 51 | 4% | 3% |
| Q | 56 | 5% | 5% |
| R | 94 | 8% | 8% |
| S | 75 | 7% | 7% |
| T | 20 | 2% | 2% |
| Household size (no. of persons), n=1139 | | | |
| 1 | 163 | 14% | |
| 2 | 359 | 32% | |
| 3 | 316 | 28% | |
| 4 | 229 | 20% | |
| 5 or above | 72 | 6% | |
| No. of members joining the study, n=1143 | | | |
| 1 | 545 | 48% | |
| 2 | 374 | 33% | |
| 3 | 129 | 11% | |
| 4 | 74 | 6% | |
| 5 or above | 21 | 2% | |
| Housing type, n=1135 | | | |
| Public housing | 311 | 27% | |
| Home ownership scheme | 202 | 18% | |
| Private housing | 618 | 54% | |
| Temporary housing | 3 | 0.3% | |
| Hotel | 1 | 0.1% | |
| With participating members born in 1986 or after, n=1143 | | | |
| No | 857 | 75% | |
| Yes | 286 | 25% | |
| With member(s) testing HBsAg positive | | | |
| No | 997 | 87% | |
| Yes | 146 | 13% | |

**Supplementary Table S4. HBsAg prevalence by age groups and gender**

| | Male | | | Female | | | Population (mid-2019)# | |
|---|---|---|---|---|---|---|---|---|
| **Age groups** | **n** | **HBsAg prevalence** | **95%CI** | **n** | **HBsAg prevalence** | **95%CI** | **Male (%)** | **Female (%)** |
| **<29** | 120 | 0.8 | (0-2.4) | 114 | 0.0 | (0-0) | 13.06 | 13.04 |
| **29-38** | 108 | 6.5 | (1.8-11.2) | 160 | 8.1 | (3.8-12.4) | 6.21 | 9.15 |
| **39-48** | 135 | 8.1 | (3.5-12.8) | 186 | 9.7 | (5.4-14) | 6.23 | 9.15 |
| **49-58** | 189 | 10.1 | (5.7-14.4) | 276 | 10.1 | (6.6-13.7) | 7.29 | 8.92 |
| **59-68** | 223 | 7.2 | (3.8-10.6) | 306 | 6.9 | (4-9.7) | 6.95 | 7.19 |
| **>68** | 130 | 10.0 | (4.8-15.2) | 123 | 6.5 | (2.1-10.9) | 5.87 | 6.96 |

#population estimates from Census and Statistics Department
https://www.censtatd.gov.hk/en/scode150.html

**Supplementary Table S5. Comparison between HBsAg negative (N=1422) and positive participants (N=105), after exclusion of single member household participants**

| | **HBsAg negative (N=1422)** | | **HBsAg positive (N=105)** | | **Odds Ratio (OR)** | | **adjusted OR^** | |
|---|---|---|---|---|---|---|---|---|
| | **n** | **%** | **n** | **%** | **OR** | **(95%C.I.)** | **aOR** | **(95%C.I.)** |
| **Socio-demographics** | | | | | | | | |
| Gender, N=1527 | | | | | | | | |
| Female | 792 | 93% | 59 | 7% | ref | | ref | |
| Male | 630 | 93% | 46 | 7% | 0.98 | (0.66-1.46) | 0.99 | (0.66-1.48) |
| Median age (IQR), N=1527 | 51 | 35-62 | 55 | 42.5-62 | 1.02* | (1.003-1.03) | / | |
| Year of birth | 1198 | 92% | 103 | 8% | ref | | | |
| <=1990 | 224 | 99% | 2 | 1% | 0.10* | (0.03-0.42) | / | |
| >1990 | | | | | | | | |
| Ethnicity, N=1520 | | | | | | | | |
| Non-Chinese | 10 | 100% | 0 | 0% | | | | |
| Chinese | 1405 | 93% | 105 | 7% | / | | / | |
| Hong Kong permanent residents, N=1519 | | | | | | | | |
| No | 30 | 86% | 5 | 14% | ref | | ref | |
| Yes | 1385 | 93% | 99 | 7% | 0.43 | (0.16-1.13) | 0.37 | (0.14-1.01) |
| Born in Hong Kong, N=1527 | | | | | | | | |
| No | 373 | 89% | 46 | 11% | ref | | ref | |
| Yes | 1049 | 95% | 59 | 5% | 0.46* | (0.3-0.68) | 0.50* | (0.33-0.76) |
| Marital status, N=1453 | | | | | | | | |
| Never married | 330 | 96% | 13 | 4% | ref | | ref | |
| Married | 1019 | 92% | 91 | 8% | 2.27* | (1.25-4.11) | 2.32* | (1.16-4.65) |
| Education level, N=1520 | | | | | | | | |
| Secondary and below | 791 | 91% | 76 | 9% | ref | | ref | |
| Post-secondary | 624 | 96% | 29 | 4% | 0.48* | (0.31-0.75) | 0.55* | (0.34-0.87) |
| **History of hepatitis or other liver diseases** | | | | | | | | |
| Liver diseases, N=1523 | | | | | | | | |
| No | 1344 | 93% | 94 | 7% | ref | | ref | |
| Yes | 74 | 87% | 11 | 13% | 2.13* | (1.09-4.14) | 1.97* | (1.004-3.86) |
| Fatty liver, N=1527 | | | | | | | | |
| No | 1353 | 93% | 95 | 7% | ref | | ref | |
| Yes | 69 | 87% | 10 | 13% | 2.06* | (1.03-4.14) | 1.92 | (0.95-3.87) |
| Cirrhosis, N=1527 | | | | | | | | |
| No | 1421 | 93% | 105 | 7% | | | | |

| | HBsAg negative (N=1422) | | HBsAg positive (N=105) | | Odds Ratio (OR) | | adjusted OR^ | |
|---|---|---|---|---|---|---|---|---|
| | **n** | **%** | **n** | **%** | **OR** | **(95%C.I.)** | **aOR** | **(95%C.I.)** |
| Yes | 1 | 100% | 0 | 0% | / | | | |
| Liver cancer, N=1527 | | | | | | | | |
| No | 1420 | 93% | 103 | 7% | ref | | ref | |
| Yes | 2 | 50% | 2 | 50% | 13.79* | (1.92-98.87) | 13.05* | (1.78-95.58) |
| Family member with HBV infection, N=1500 | | | | | | | | |
| No | 1138 | 95% | 60 | 5% | ref | | ref | |
| Yes | 254 | 86% | 43 | 14% | 3.21* | (2.12-4.86) | 3.49* | (2.25-5.42) |
| Not sure | 3 | 60% | 2 | 40% | 12.64* | (2.07-77.1) | 10.21* | (1.66-62.77) |
| **Community exposure risk** | | | | | | | | |
| History of illicit drug use, N=1459 | | | | | | | | |
| No | 1354 | 93% | 105 | 7% | | | | |
| Yes | 0 | 0% | 0 | 0% | / | | / | |
| Sex experience, N=1463 | | | | | | | | |
| No | 211 | 96% | 8 | 4% | ref | | ref | |
| Yes | 1147 | 92% | 97 | 8% | 2.23* | (1.07-4.66) | 2.08 | (0.95-4.57) |
| No. of lifetime sex partners, N=1430 | | | | | | | | |
| None | 211 | 96% | 8 | 4% | ref | | ref | |
| 1 | 824 | 92% | 71 | 8% | 2.27* | (1.08-4.79) | 2.09 | (0.92-4.74) |
| 2 to 5 | 256 | 92% | 22 | 8% | 2.27 | (0.99-5.2) | 2.26 | (0.97-5.26) |
| 6 to 10 | 27 | 93% | 2 | 7% | 1.95 | (0.39-9.68) | 2.01 | (0.4-10.12) |
| 11 or more | 9 | 100% | 0 | 0% | / | | | |
| **Risk exposure in the healthcare setting** | | | | | | | | |
| Frequency of receiving intravenous injection, N=1506 | | | | | | | | |
| Never | 911 | 95% | 53 | 5% | ref | | ref | |
| Ever | 491 | 91% | 51 | 9% | 1.79* | (1.2-2.66) | 1.70* | (1.14-2.54) |
| Frequency of blood transfusion, N=1499 | | | | | | | | |
| Never | 1211 | 93% | 88 | 7% | ref | | ref | |
| Ever | 183 | 92% | 17 | 9% | 1.28 | (0.74-2.2) | 1.16 | (0.67-2) |
| History of dialysis, N=1499 | | | | | | | | |
| Never | 1390 | 93% | 105 | 7% | | | | |
| Ever | 4 | 100% | 0 | 0% | / | | / | |
| History of surgery, N=1502 | | | | | | | | |
| Never | 791 | 93% | 58 | 7% | ref | | ref | |
| Ever | 607 | 93% | 46 | 7% | 1.03 | (0.69-1.54) | 0.90 | (0.6-1.37) |

| | HBsAg negative (N=1422) | | HBsAg positive (N=105) | | Odds Ratio (OR) | | adjusted OR^ | |
|---|---|---|---|---|---|---|---|---|
| | **n** | **%** | **n** | **%** | **OR** | **(95%C.I.)** | **aOR** | **(95%C.I.)** |
| **Vaccination history** | | | | | | | | |
| Self-reported HBV vaccination, N=1517 | | | | | | | | |
| No | 847 | 90% | 96 | 10% | ref | | ref | |
| Yes | 565 | 98% | 9 | 2% | 0.14* | (0.07-0.28) | 0.14* | (0.07-0.29) |
| HBV childhood immunisation in Hong Kong#, N=1527 | | | | | | | | |
| Unlikely | 1108 | 92% | 102 | 8% | ref | | ref | |
| Likely | 314 | 99% | 3 | 1% | 0.10* | (0.03-0.33) | 0.07* | (0.02-0.24) |

# Born in Hong Kong in or after 1984, or migrate to Hong Kong at the age 12 or below
^age and household member testing HBsAg positive as confounders in multivariable logistic regression
*p<0.05

**Supplementary Table S6. Characteristics of participants protected from hepatitis B infection (HBsAg-/Anti-HBs+) (n=1037) and those with current infection (HBsAg+/Anti-HBs-) (n=147)**

| | currently protected | | current infection | |
|---|---|---|---|---|
| | n | % | n | % |
| **Socio-demographics** | | | | |
| Gender | | | | |
| Female | 587 | 57% | 83 | 56% |
| Male | 450 | 43% | 64 | 44% |
| Median age (IQR) | 54 | 45-63 | 55 | 42-63 |
| Year of birth group | | | | |
| In or before 1950 | 108 | 10% | 16 | 11% |
| 1951-1960 | 281 | 27% | 34 | 23% |
| 1961-1970 | 241 | 23% | 46 | 31% |
| 1971-1980 | 181 | 17% | 30 | 20% |
| 1981-1990 | 140 | 14% | 19 | 13% |
| After 1990 | 86 | 8% | 2 | 1% |
| Ethnicity, n=1178 | | | | |
| Non-Chinese | 3 | 0.3% | 0 | 0% |
| Chinese | 1029 | 99.7% | 146 | 100% |
| Hong Kong permanent residents, n=1177 | | | | |
| No | 26 | 3% | 6 | 4% |
| Yes | 1006 | 97% | 139 | 96% |
| Born in Hong Kong | | | | |
| No | 325 | 31% | 70 | 48% |
| Yes | 712 | 69% | 77 | 52% |
| Marital status, n=1182 | | | | |
| Never married | 211 | 20% | 24 | 16% |
| Widowed | 54 | 5% | 7 | 5% |
| Separated | 4 | 0% | 0 | 0% |
| Divorced | 71 | 7% | 18 | 12% |
| Married | 664 | 64% | 97 | 66% |
| NA, aged 15 or below | 32 | 3% | 0 | 0% |
| **HBV vaccination status** | | | | |
| Self-report HBV vaccination | | | | |
| No | 556 | 54% | 136 | 93% |
| Yes | 475 | 46% | 11 | 7% |
| Childhood immunization in Hong Kong# | | | | |
| Unlikely | 886 | 85% | 141 | 96% |
| Likely | 151 | 15% | 6 | 4% |

# Born in Hong Kong in or after 1984, or migrate to Hong Kong at the age 12 or below

**Supplementary Table S7. Characteristics of participants who tested anti-HBs+ and HBsAg- (immune), HBsAg+ and anti-HBs- (chronically infected), and HBsAg- and anti-HBs- (susceptible), after the exclusion of cases with unclear status (HBsAg-, anti-HBc+, and anti-HBs-)**

| | Immune (n=1037) | | Chronically infected (n=155) | | Susceptible (n=872) | | Multivariable multinominal logistic regression (immune as reference) † infected | | susceptible | |
|---|---|---|---|---|---|---|---|---|---|---|
| | n | % | n | % | n | % | aOR | 95%CI | aOR | 95%CI |
| **Socio-demographics** | | | | | | | | | | |
| Gender, N=2064 | | | | | | | | | | |
| Female | 587 | 57% | 88 | 57% | 485 | 56% | 0.98 | (0.7-1.39) | 0.99 | (0.82-1.19) |
| Male | 450 | 43% | 67 | 43% | 387 | 44% | ref | | ref | |
| Median age (IQR), N=2064 | 54 | (42-63) | 56 | (45-63) | 53 | (35-63) | OR= 1.01 | (0.999-1.02) | OR= 0.99* | (0.99-0.997) |
| Year of birth group | | | | | | | | | | |
| In or before 1960 | 389 | 38% | 54 | 35% | 305 | 35% | OR= 5.97* | (1.43-24.96) | OR= 0.42* | (0.31-0.57) |
| 1961-1970 | 241 | 23% | 49 | 32% | 177 | 20% | OR= 8.74* | (2.08-36.72) | OR= 0.40* | (0.29-0.55) |
| 1971-1980 | 181 | 17% | 31 | 20% | 125 | 14% | OR= 7.36* | (1.72-31.48) | OR= 0.37* | (0.26-0.53) |
| 1981-1990 | 140 | 14% | 19 | 12% | 106 | 12% | OR= 5.84* | (1.33-25.68) | OR= 0.41* | (0.28-0.59) |
| After 1990 | 86 | 8% | 2 | 1% | 159 | 18% | ref | | ref | |
| Ethnicity, N=2052 | | | | | | | | | | |
| Non-Chinese | 3 | 0% | 0 | 0% | 9 | 1% | / | | 3.58 | (0.96-13.39) |
| Chinese | 1029 | 100% | 154 | 100% | 857 | 99% | | | ref | |
| Hong Kong permanent residents, N=2052 | | | | | | | | | | |
| No | 26 | 3% | 6 | 4% | 9 | 1% | 1.69 | (0.68-4.21) | 0.37* | (0.17-0.8) |
| Yes | 1006 | 97% | 147 | 96% | 858 | 99% | ref | | ref | |
| Born in Hong Kong, N=2063 | | | | | | | | | | |
| No | 325 | 31% | 76 | 49% | 194 | 22% | 2.03* | (1.44-2.86) | 0.66* | (0.54-0.82) |
| Yes | 712 | 69% | 79 | 51% | 677 | 78% | ref | | ref | |
| Ever married, N=1987 | | | | | | | | | | |
| No | 211 | 21% | 24 | 16% | 260 | 31% | 0.88 | (0.55-1.41) | 1.28* | (1.004-1.64) |
| Yes | 793 | 79% | 130 | 84% | 569 | 69% | ref | | ref | |
| Education level, N=2057 | | | | | | | | | | |
| Secondary and below | 582 | 56% | 109 | 70% | 486 | 56% | 1.76* | (1.22-2.54) | 1.04 | (0.87-1.25) |
| Post-secondary | 451 | 44% | 46 | 30% | 383 | 44% | ref | | ref | |
| History of liver diseases, N=2059 | | | | | | | | | | |
| No | 978 | 94% | 134 | 87% | 815 | 94% | 0.43* | (0.25-0.73) | 0.80 | (0.54-1.17) |
| Yes | 58 | 6% | 20 | 13% | 54 | 6% | ref | | ref | |
| any member tested HBsAg positive | | | | | | | | | | |
| No | 693 | 89% | 89 | 85% | 582 | 91% | 0.63 | (0.35-1.13) | 1.36 | (0.94-1.95) |
| Yes | 84 | 11% | 16 | 15% | 58 | 9% | ref | | ref | |
| **HBV vaccination status** | | | | | | | | | | |
| Self-report HBV vaccination, N=2052 | | | | | | | | | | |
| No | 556 | 54% | 143 | 92% | 625 | 72% | 9.53* | (5.22-17.42) | 2.59* | (2.11-3.17) |
| Yes | 475 | 46% | 12 | 8% | 241 | 28% | ref | | ref | |
| HBV childhood immunization in Hong Kong#, N=2064 | | | | | | | | | | |
| Unlikely | 886 | 85% | 149 | 96% | 674 | 77% | 2.74* | (1.01-7.42) | 1.18 | (0.8-1.73) |
| Likely | 151 | 15% | 6 | 4% | 198 | 23% | ref | | ref | |

# Born in Hong Kong in or after 1984, or migrate to Hong Kong at the age 12 or below
†adjusted by variable of year of birth after 1990 (vs in or before 1990) in multivariable multinominal logistic regression
*p<0.05

**Supplementary Fig. 1 Timeline of the evolvement of the universal hepatitis B vaccination programme in Hong Kong**

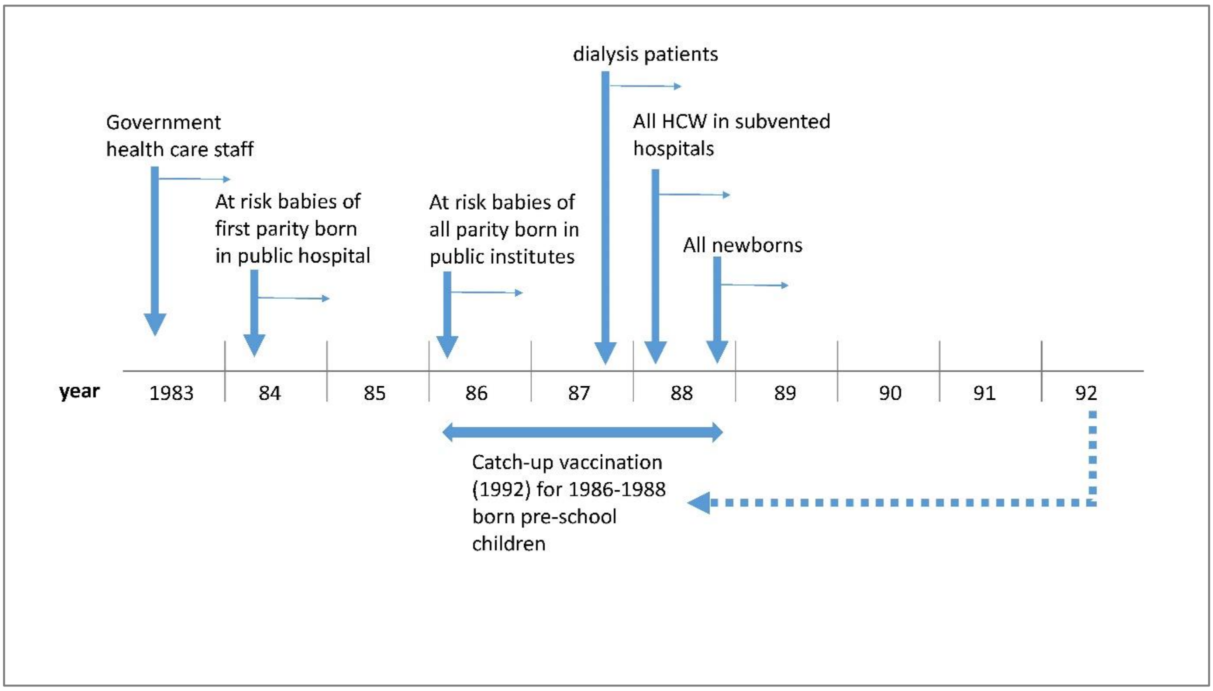

**Supplementary Fig. 2. Map showing (A). the distribution of participants' residential locations by HBsAg test result (positive and negative); (B) proportion of household index participants testing HBsAg positive; and (C) anti-HBs positive in 18 districts**

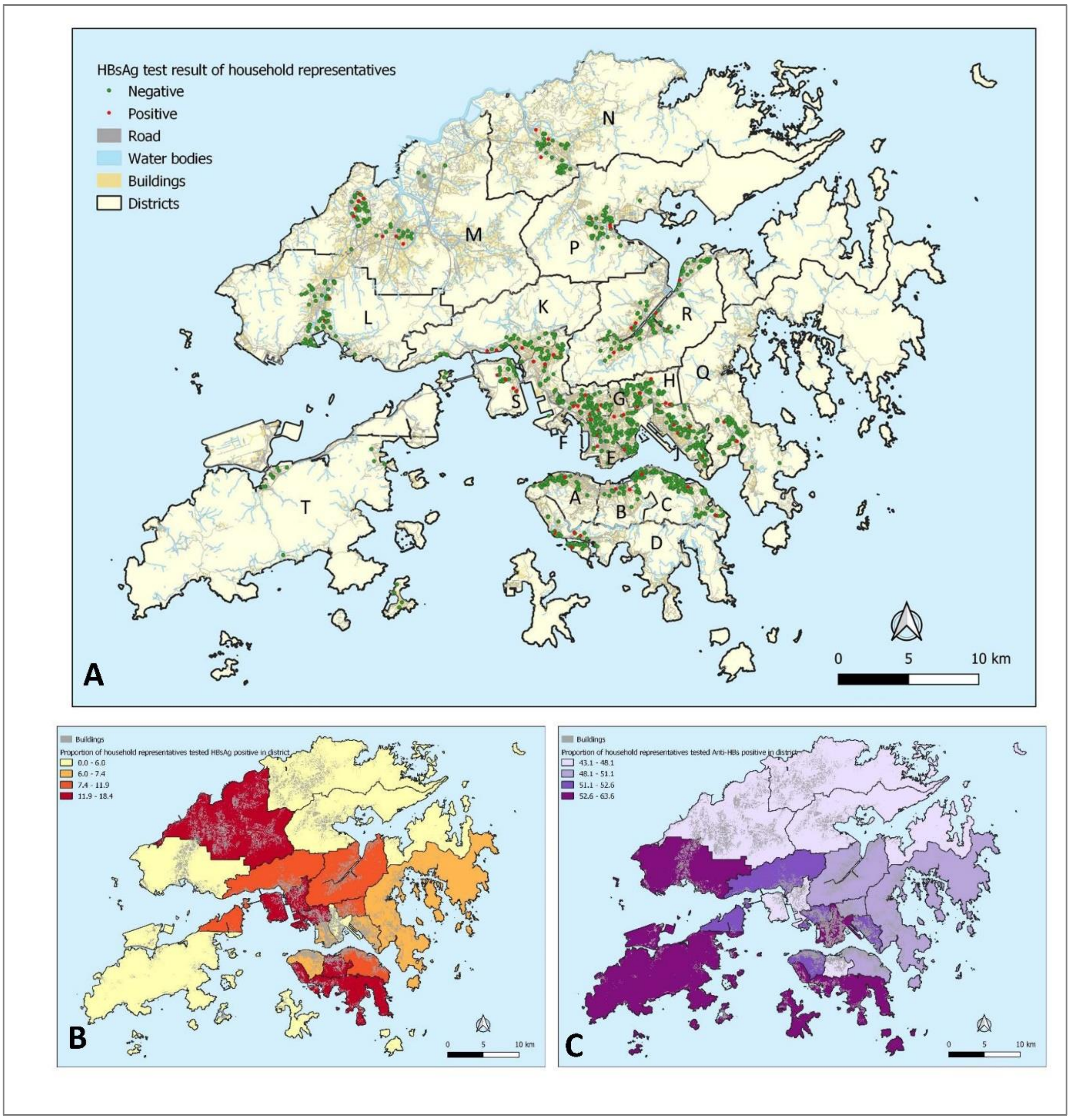

Supplement: Supplementary file 1 [file S095026882300002Xsup001.pdf]
